# *Pneumocystis Jirovecii* Pneumonia in a Kidney Transplant Recipient 13 Months after Transplantation: A Case Report and Literature Review

**DOI:** 10.15388/Amed.2020.28.1.5

**Published:** 2021-01-25

**Authors:** Dominykas Varnas, Augustina Jankauskienė

**Affiliations:** Vilnius University Hospital Santaros Klinikos, Pediatric Center, LT-08406 Vilnius, Lithuania Vilnius University, Institute of Clinical Medicine, Vilnius, Lithuania; Vilnius University Hospital Santaros Klinikos, Pediatric Center, LT-08406 Vilnius, Lithuania Vilnius University, Institute of Clinical Medicine, Vilnius, Lithuania

**Keywords:** *Pneumocystis jirovecii*, *Pneumocystis* pneumonia, Kidney transplant recipients, case report

## Abstract

**Summary. Background.:**

*Pneumocystis jirovecii* pneumonia (PCP) is an opportunistic and prevalent fungal infection in immunocompromised hosts, including patients after kidney transplantation (KTx). It is a life threatening infection. While with effective prophylaxis it became less common, it still remains an issue among solid organ transplant (SOT) recipients during the first year. There are no specific clinical signs for PCP. Computed tomography (CT) is a better method for detecting PCP, but definite diagnosis can only be made by identification of the microorganism either by a microscopy or by a polymerase chain reaction (PCR).

**Clinical case.:**

We present a case of a 17 year old with severe PCP 13 months after KTx followed by reduction in kidney function and respiratory compromise. The pathogen was detected by PCR from bronchoalveolar lavage fluid (BALF) and patient was treated successfully with trimethoprim-sulfamethoxazole (TMPSMX). Patient’s condition, respiratory status and kidney function gradually improved. Our presented case is unusual because patient had no known risk factors for PCP and he was more than one year after KTx, what is considered rare. In addition patient and his parents delayed in notifying the treating physician about ongoing symptoms because did not deem them important enough.

**Conclusions.:**

Clinicians treating patients in risk groups for PCP must always remain vigilant even in era of effective prophylaxis. The vigilance should also extend to the patient and patient’s family.

## Abstract (lt)

***Pneumocystis jirovecii* pneumonija inksto transplantato recipientui praėjus 13 mėnesių po transplantacijos: klinikinis atvejis ir literatūros apžvalga:**

**Santrauka. Apžvalga.**

*Pneumocystis jirovecii* pneumonija (PJP) yra oportunistinė grybelinė infekcija, paplitusi tarp pacientų, kurių imuninė sistema yra susilpnėjusi, įskaitant pacientus po inkstų transplantacijos (Tx). Tai gyvybei pavojinga infekcija. Nors dėl veiksmingos profilaktikos infekcija tapo mažiau paplitusi, ji vis dar aktuali dėl galimo plitimo tarp solidinių organų transplantacijos recipientų. Konkrečių PJP klinikinių požymių nėra. Kompiuterinė tomografija yra geresnis PJP nustatymo metodas, tačiau neklystamai diagnozuoti galima tik identifikuojant mikroorganizmą mikroskopu arba polimerazės grandininės reakcijos (PGR) metu.

17 metų pacientui diagnozuota sunki PJP, inkstų funkcijos pablogėjimas, kvėpavimo nepakankamumas, praėjus 13 mėnesių po inkstų Tx. Ligos sukėlėjas nustatytas iš bronchoalveolinio lavažo skysčio PGR metodu ir pacientas sėkmingai gydytas trimetoprimu ir sulfametoksazolu. Paciento bendra būklė, kvėpavimo funkcija ir inkstų funkcija pamažu pagerėjo. Pateiktas atvejis yra neįprastas, nes pacientas neturėjo jokių žinomų PJP rizikos veiksnių ir buvo daugiau nei vieneri metai praėję po inkstų Tx. Galimai turėjo įtakos paciento ir jo tėvų vėlyvas kreipimasis, nes jie nesuprato pradinių simptomų prasmės ir reikšmingumo.

**Išvados.**

Gydytojai, gydantys PJP rizikos grupių pacientus, visada turi būti budrūs net ir veiksmingos profilaktikos laikais. Pacientai ir jų artimieji taip pat turi išlikti sąmoningi ir atidūs, tai leistų anksti pastebėti simptomus.

**Raktažodžiai:** *Pneumocystis jirovecii, Pneumocystis* pneumonija, inkstų transplanto recipientai, atvejo aprašymas.

## Introduction

*Pneumocystis jirovecii* pneumonia is an opportunistic and life-threatening fungal infection that is prevalent among immunocompromised hosts [1]. While most humans are infected with *P. jirovecii* by 2–3 years of age, it is asymptomatic in immunocompetent children [2,3]. Risk factors for developing a clinically significant Pneumocystis pneumonia include: acquired immunodeficiency syndrome (AIDS), glucocorticoid and other immunosuppresive agent treatment, allogeneic hematopoietic stem cell transplantation, SOT and congenital immunodeficiency syndromes [4].

In pre-chemoprophylaxis era the over-all incidence of *P. jirovecii *infection among SOT recipients varied in the range of 5–15% [5]. After the introduction of chemoprophylaxis, the incidence of PCP has decreased dramatically [6], although it still remains an important causative pathogen among SOT recipients [7]. However, the survival of non-HIV patients with PCP is worse than those with AIDS and reaches up to 50% even with adequate therapy [8].

Here we present a case of a late-onset PCP in an adolescent patient more than a year after KTx, with no known significant risk factors before the beginning of illness.

## Clinical Case

The patient is a 17 year old boy with a history of a congenital vesicoureteral reflux and other congenital abnormalities of kidney and urinary tract (CAKUT) which gradually caused nephrosclerosis, secondary hypertension and chronic kidney disease (CKD). In April, 2019 he started peritoneal dialysis (PD) because of CKD stage 5. In June, 2019 he received a deceased donor kidney transplant (from a 58 year old female, cause of death – intracerebral hemorrhage. CMV +, EBV +, hepatitis markers – negative, CMV donor + /recipient –). Before KTx induction therapy consisted of Basiliximab – interleukin 2 receptor antibody (IL2Ra) 20 mg IV, mycophenolate mofetil (MMF) 750 mg PO and tacrolimus 3 mg PO. Intraoperatively methylprednisolone 500 mg IV was administered. After KTx patient underwent maintenance therapy with tacrolimus 3 mg PO once daily (o.d.), MMF 750 mg PO twice daily, methylprednisolone 48 mg PO Prophylaxis for Cytomegalovirus (CMV) with valganciclovir 450 mg PO for 6 months as high risk and prophylaxis for *Pneumocystis jirovecii* TMPSMX 80/400 mg PO was given for 3 months (up to September, 2019). Additionally, patient received treatment with antihypertensives and anticoagulants.

The post-transplant period was mostly uncomplicated with good diuresis, patient had no rejection episodes. Tacrolimus, other immunosuppressant and antihypertensive treatment was tailored a few times in order to achieve either required drug serum concentrations or clinical efficacy, graft function was relatively stable, eGFR ranging from 44,1 to 51,4 mL/min/1.73m². Darbopoetin alfa 20 μg once every two weeks s/c and folic acid was started due to secondary anemia and continued for 6 months. In July, 2019 the first graft biopsy was performed due to proteinuria and poorly controlled hypertension. Results showed focal mesangioproliferative changes without immunoreactant deposition. Immunosuppresive treatment with tacrolimus and antihypertensive treatment was intensified

In September, 2019 the patient was hospitalised due to leukopenia, neutropenia, diarrhea, loss of apetite. His blood tests showed elevated CMV viremia (up to 8811 copies/ml, while on previous outpatient visits no viremia was detected). MMF was discontinued and valgancivlovir dose was increased up to 450 mg twice daily, for 2 weeks until viral load became undetectable. Patient had fungal toenail infection, topical terbinafine and naftifine were prescribed. Valganciclocvir was discontinued in December, 2019, after completion of 6 months of post-KTx prophylaxis. The last outpatient visit was 12 days before the case of PCP. During the visit the patient had no complaints, his physical examination was unremarkable, graft function was stable with eGFR of 41,4 mL/min/1.73m² and low CMV viremia of 25 copies/ml (which was below the significant threshold). His immunosuppressive regimen consisted of tacrolimus 6 mg PO o.d., methylprednisolone 8 mg PO o.d. and MMF 500 mg PO twice daily and were not tailored recently.

At the end of June, 2020 patient was admitted to the hospital due to the subfebrile temperature, dry cough, shortness of breath for about 1 week and general fatigue for more than 3 weeks during physical activity. Important to note that additional information about earlier onset of patient’s complaints was explicated only after the patient was hospitalized, because at last outpatient visit they did not inform about complaints of fatigue. Patient also lost 4 kg down to 46 kg of weight in the last month. On physical examination the patient was tachycardic (HR – 128 bpm), oxygen saturation (SpO_2_) was 89% and a subfebrile temperature of 37.2^o^C. Patient required 2–3 L/min of oxygen to maintain oxygen saturation above 92%. Blood pressure (BP) was 124/80mmHg. There was a reddish, dry maculopapular rash on the upper side of the trunk and around the neck and signs of toenail fungus were seen. Lung auscultation was normal. PCR tests for SARS-CoV-2 from nasopharyngeal swabs were negative twice. No other remarkable findings.

Initial laboratory tests showed decreasing eGFR down to 32 mL/min/1.73m². C-reactive protein (CRP) was elevated – 79.2 mg/l, tacrolimus concentration – 5.7 ng/ml. Complete blood count, albumin, liver enzymes and electrolytes were normal (laboratory tests are presented in Table 1). Tuberculosis was excluded with both negative Tuberculin skin test (TST) and Interferon-gamma release assay. Atypical bacterial infections were excluded with negative serological testing and negative nasopharyngeal swab PCR. Blood culture was taken. Both EBV and CMV blood tests for PGR were taken and CMV viremia was present at 51 copies/ml.

**Figure 1.**
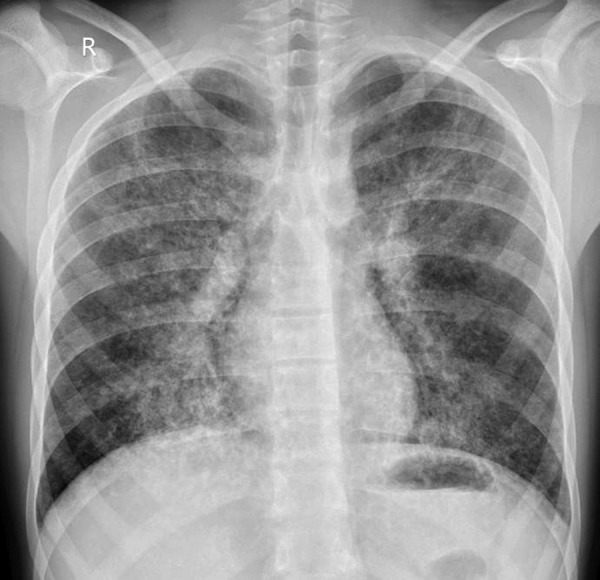
Chest radiograph. Fine bilateral interstitial infiltrates with interstitial edema.

Chest radiograph (Figure 1) revealed fine bilateral interstitial infiltrates with interstitial edema.

Immunosupressive and antihypertensive treatment was initially unchanged. Topical antifungal treatment was prescribed for suspected fungal etiology of toenail and skin lesions while microbiological tests were ongoing. O_2_ flow through face mask of 2 l/min was continued.

**Figure 2.**
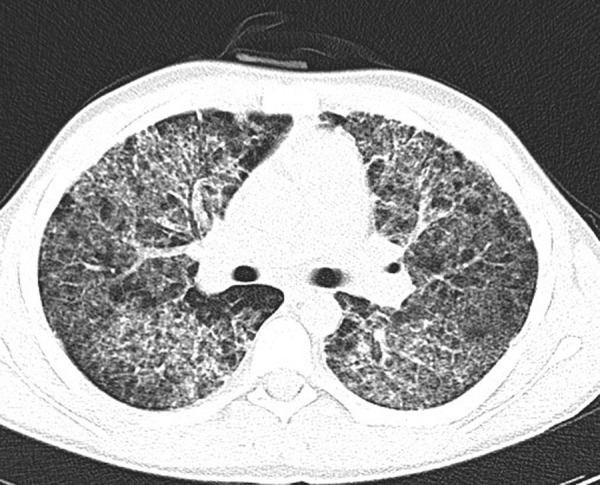
Chest CT. Bilateral ground glass opacities and diffuse centrilobular and peribronchovascular consolidation foci.

Chest CT scan (Figure 2) showed bilateral ground glass opacities, pneumatoceles in basal segments and diffuse varying in size centrilobular and peribronchovascular consolidation foci.

On the second day of hospitalisation, while the workup was still in process and bronchoscopy for acquisition of BALF was in line, patient’s condition deteriorated, resulting in increased breathing rate, dyspnea and supplemental oxygen demand of up to 12 l/min in order to keep SpO_2_ ≥92%. It was followed by a decrease of kidney function down to eGFR of 27,1 mL/min/1.73m². Patient was empirically started on TMP-SMX 320 mg IV o.d., Fluconasole 100 mg IV o.d., Sol. Ganciclovir 115mg IV o.d. MMF dose was reduced to 250 mg twice daily, which was later temporarily discontinued. A bronchoscopy was performed and a BALF sample tested positive for *P. jirovecii. *CMV (3575 copies/ml) and EBV (70000 copies/ml) were detected too. In addition, immunoglobulin G (IgG) was found to be significantly lower – 2,16 g/l, so an IV infusion of 20g IgG was administered.

**Figure 3.**
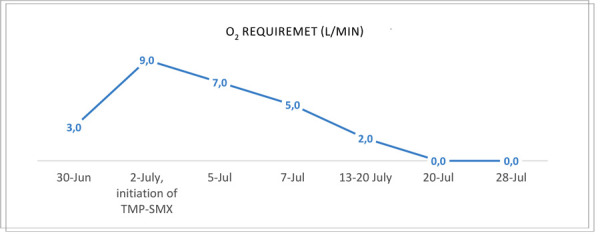
O_2_ therapy requirement during treatment in order to keep Sp O_2_ ≥92%.

Over the next days the patient’s condition improved, with a reduction of O_2_ therapy (Figure 3) and a gradual resolution of symptoms – patient reported no fatigue, better general well-being and only occasional coughing. Slight improvement in kidney function was observed with eGFR of 30,9 mL/min/1.73m² and patient was discharged 2 and a half weeks later after he received IV TMP-SMX for 18 days, and IV ganciclovir for 1 week. Later valganciclovir PO was continued for a total of 1 month and TMP-SMX at a lower dose of 480 mg PO was continued for 3 months in total.

At follow-up his condition has progressively improved, with no signs of infection or respiratory distress, with a markedly improved kidney function and normal inflammation markers as seen in Table 1.

**Table 1.** Laboratory tests

Date	Initial June 30	At the start of IV TMP-SMX July 2	Before discharge July 20	1 week after discharge July 28
WBC (x10^9^/L)	5.57	4.37	7.21	8.76
Lymphocites (x10^9^/L)	2.77	2.05	4.92	6.0
Neutrophils (x10^9^/L)	2.07	1.72	1.67	2.27
Platelets (x109/L)	437	376	419	273
eGFR (mL/min/1.73 m²)	32	27,1	30,9	41,2
pCO_2_* (mmHg)	35.5	42.2	46.4	49.3
CRP (mg/l)	79.2	84.4	5.64	0.05

## Discussion

PCP is a relevant and life-threatening infection after kidney transplantation. Before the broad implementation of prophylaxis the infection developed in up to 15% of SOT recipients [9]. Even though its frequency has markedly decreased with a wide-spread prophylaxis with TMP-SMX, it still remains an issue with an incidence of up to 2,5%. The occurrence of PCP in SOT patients gradually increases for those with risk factors (highly immunosuppressed patients, infected with CMV, prolonged neutropenia and higher-dose corticosteroid therapy). Reinstitution of prophylaxis is usually recommended in such cases [10].

In our case a relevant factor was that kidney donor was CMV-positive and the recipient was CMV-negative. Current data shows that present or even past episode of CMV infection (more so because of CMV reactivation than *de novo* infection) is a causal risk factor for PCP [11, 12]. Interestingly, biological induction immunosuppressive regimen choice was found not to be a risk factor for opportunistic infections [13].

Moreover, a systematic review showed that IL2Ra, used as an addition or alternative to induction therapy, did not demonstrate an increase in total serious infections when compared to a placebo and no treatment groups [14]. The picture is not clear-cut as some studies point to increased risk of CMV, EBV or bacterial infections in patients who received induction with biological therapy, but not specifically for PCP [15, 16]. There is no reliable evidence to suggest that PCP can be associated with IL2Ra induction therapy in our case. 

Another possible risk factor for development of infection after kidney transplantation is secondary hypogammaglobulinemia (HGG). HGG is a common complication after SOT occurring in up to 45% of patients [17] during the first post-transplantation year. There are different reasons for secondary HGG after SOT, but in our presented case the immunosupressive treatment (namely MMF and corticosteroids as main culprits) is the most likely cause [18]. Important to note that while more data supports a positive relation between hypogammaglobulinemia and infections [19, 20], some studies concluded otherwise [21]. Nonetheless, there is still no consensus on preventive measures for infections regarding hypogammaglobulinemia in kidney transplant recipients [21, 22, 23]. In our case of secondary HGG the decision was made to administer IV IgG in order to correct a substantial deficiency and thus possibly improve patient’s outcome.

Regarding PCP prophylaxis Current Kidney Disease: Improving Global Outcomes (KDIGO) guidelines recommend TMP-SMX as a first-line therapy for 3–6 months after KTx and at least 6 weeks during and after treatment for acute rejection. Alternative therapies with dapsone, aerosolized pentamidine or atovaquon should be considered in the event of allergic reactions or severe side effects [24].

In case of PCP, treatment with TMP-SMX is also considered as a first-line therapy with 15 to 20 mg/kg IV, switching to oral after clinical improvement. Second-line therapy for severe cases would be pentamide 4 mg/kg IV o.d., though with comparable efficacy but worse safety profile [25, 26, 27]. Primaquine plus Clindamycin for severe cases and Atovaquone or Dapsone/TMP for mild to moderate cases are third-line options that can be considered for patients who do not tolerate the above treatments or show no clinical improvement. However, most of the data about treatment regimens is based on published literature in HIV-infected children group, derived from adolescent–adult studies or have no available data in pediatric group whatsoever [28, 29].

Both in our case and in literature clinical manifestation of PCP is described as not specific. The classical presentation includes nonproductive cough, dyspnea, and fever, which gradually progresses into respiratory failure. Rarely patients may be asymptomatic or may present with weight loss, chest pain, hemoptysis [30]. Physical examination might be unremarkable, but hypoxemia and tachypnea are more common, while discrete crackles are only occasionally present [1,30,31]. Clinical presentation and disease progression differ depending on the etiology: HIV-positive patients tend to have a more indolent, protracted disease course and more favourable outcomes compared to non-HIV patients [32, 33]. Presented case reaffirms this. In 2020 summer we differentiated with Covid-19 infection as well because of difficulty to breath, saturation drop down and fever. Though initial symptoms might have been weight loss or general fatigue during physical activity. Progression of mild respiratory symptoms towards respiratory failure was rapid, in over one week.

Chest radiograph may be normal or reveal diffuse bilateral interstitial pulmonary infiltrates that are an unspecific sign for a PCP [34]. High-resolution CT scan is a more sensitive and specific method for detecting PCP. In our case it showed bilateral ground glass opacities, pneumatoceles in basal segments and diffuse varying in size centrilobular and peribronchovascular consolidation foci as typically described in the literature as well [1,34].

Studies are ongoing regarding the serological testing. Role of serum levels of lactate dehydrogenase (LDH), β-D-glucan and KL-6 have been evaluated in the diagnosis of PCP. However, no standard for serological diagnosis of PCP exists to this day [35, 36].

The definite diagnosis of PCP requires an identification of the microorganism either by a microscopy or by PCR with the sample taken from BALF [37]. Due to higher sensitivity (98.3%) and specificity (91.0%) PCR is increasingly used as a diagnostic method for PCP. Moreover, microscopy has remarkably lower sensitivity for use in HIV-uninfected patients, thus making the PCR method more preferable [38]. A clinical challenge in making diagnosis remains, since a definite cut-off values for *P. jirovecii*, CMV or EBV load do not exist [39] and vary greatly between different institutions [40, 41]. The reported case was no exception because viral loads for CMV (3575 copies/ml) found in BALF would be considered in the “gray” area by most clinicians, and for *P. jirovecci* only qualitative analysis was made giving a positive result. The diagnosis was made because of weight loss, fatigue, shortness of breath, saturation drop down, typical results of CT scan and a postive result of P. *jirovecci *in BALF.

Occurrence of PCP later than 1 year after SOT with effective prophylaxis is rare, but cases even up to 13 years after KTx [42] can be found. [43]. The presented case of PCP in an adolescent patient developed a bit more than a year after KTx. In addition, our patient is unusual because his graft function was relatively stable, he had no rejection episodes, no history of respiratory tract infections generally in his life.

In our case the patient addressed the doctor quite late, despite fatigue for about 3 weeks and difficulty to breathe for about 1 week, which they did not evaluate as important. Patient education remains important. In some cases usage of pulse oximeter could be recommended in order to self-monitor respiratory complaints.

## Conclusions

We present a case of PCP in a 17 year old boy 13 months after KTx, though he had no obvious PCP risk factors and has successively completed his prophylaxis according to KDIGO guidelines. Clinicians treating patients in risk groups for PCP must remain vigilant even in the prophylaxis era. Patients and their families must also remain alert to report symptoms early.
